# ERF5 and ERF6 Play Redundant Roles as Positive Regulators of JA/Et-Mediated Defense against *Botrytis cinerea* in Arabidopsis

**DOI:** 10.1371/journal.pone.0035995

**Published:** 2012-04-26

**Authors:** Caroline S. Moffat, Robert A. Ingle, Deepthi L. Wathugala, Nigel J. Saunders, Heather Knight, Marc R. Knight

**Affiliations:** 1 School of Biological and Biomedical Sciences, Durham Centre for Crop Improvement Technology, Durham University, Durham, United Kingdom; 2 Department of Environment and Agriculture, Australian Centre for Necrotrophic Fungal Pathogens, Curtin University, Perth, Australia; 3 Department of Molecular and Cell Biology, University of Cape Town, Rondebosch, South Africa; 4 Department of Crop Science, University of Ruhuna, Kamburupitiya, Sri Lanka; 5 Sir William Dunn School of Pathology, University of Oxford, Oxford, United Kingdom; National Taiwan University, Taiwan

## Abstract

The ethylene response factor (ERF) family in *Arabidopsis thaliana* comprises 122 members in 12 groups, yet the biological functions of the majority remain unknown. Of the group IX ERFs, the IXc subgroup has been studied the most, and includes ERF1, ERF14 and ORA59, which play roles in plant innate immunity. Here we investigate the biological functions of two members of the less studied IXb subgroup: ERF5 and ERF6. In order to identify potential targets of these transcription factors, microarray analyses were performed on plants constitutively expressing either *ERF5* or *ERF6*. Expression of defense genes, JA/Et-responsive genes and genes containing the GCC box promoter motif were significantly upregulated in both ERF5 and ERF6 transgenic plants, suggesting that ERF5 and ERF6 may act as positive regulators of JA-mediated defense and potentially overlap in their function. Since defense against necrotrophic pathogens is generally mediated through JA/Et-signalling, resistance against the fungal necrotroph *Botrytis cinerea* was examined. Constitutive expression of *ERF5* or *ERF6* resulted in significantly increased resistance. Although no significant difference in susceptibility to *B. cinerea* was observed in either *erf5* or *erf6* mutants, the *erf5 erf6* double mutant showed a significant increase in susceptibility, which was likely due to compromised JA-mediated gene expression, since JA-induced gene expression was reduced in the double mutant. Taken together these data suggest that ERF5 and ERF6 play positive but redundant roles in defense against *B. cinerea*. Since mutual antagonism between JA/Et and salicylic acid (SA) signalling is well known, the UV-C inducibility of an SA-inducible gene, *PR-1*, was examined. Reduced inducibilty in both *ERF5* and *ERF6* constitutive overexepressors was consistent with suppression of SA-mediated signalling, as was an increased susceptibility to avirulent *Pseudomonas syringae*. These data suggest that ERF5 and ERF6 may also play a role in the antagonistic crosstalk between the JA/Et and SA signalling pathways.

## Introduction

Ethylene response factors (ERFs) are members of the AP2/ERF superfamily, one of the largest families of plant transcription factors. The AP2/ERF superfamily is defined by the presence of the highly conserved AP2/ERF DNA-binding domain, consisting of approximately 60 to 70 amino acids [1]. In Arabidopsis, the AP2/ERF superfamily consists of 147 genes, of which 122 are members of the ERF family which contain a single AP2/ERF domain [2]. The ERF family members can be further divided into 12 groups based on the amino acid alignments of the AP2/ERF domains. ERF proteins are able to bind the GCC box (AGCCGCC), a short *cis*-acting element found in the promoters of many jasmonic acid (JA)/ethylene (Et)-inducible and pathogenesis-related (*PR*) genes [3], and can positively or negatively regulate transcription. For example, transient expression analyses in Arabidopsis leaves revealed that AtERF1, ERF2 and ERF5 function as activators of GCC box-mediated transcription, whilst ERF3, ERF4 and ERF7 act as repressors [4], [5].

A wide range of biological functions have been described for ERF family proteins. ERF proteins are involved in the transcriptional regulation of various responses to environmental stimuli. Several ERF transcriptional activators confer enhanced disease resistance when overexpressed and compromised resistance when disrupted. Overexpression of the transcriptional activators, *ERF1* and *ERF2*, up-regulated defense gene transcript levels (*PDF1.2* and *b-CHI*) and increased resistance to the necrotrophic pathogen *Fusarium oxysporum* [6]–[8], whilst a T-DNA insertion mutant of the transcriptional activator *ERF14* displayed impaired induction of defense gene expression (*PDF1.2* and *b-CHI*) and increased susceptibility to infection by *F. oxysporum* [9]. Conversely, mutant plants of the transcriptional repressor *ERF4* exhibited increased *PDF1.2* levels and enhanced resistance to *F. oxysporum,* whilst *ERF4-*overexpressors were more susceptible to infection by this pathogen [8], [9]. ERF proteins also play a role in a variety of developmental processes such as cell expansion, leaf petiole development and some are able to mediate the response to cytokinin [10]–[12].

Presumably reflecting their roles in stress tolerance, expression of many *ERF* genes is regulated in response to environmental stress, although their patterns vary. Regulation by disease-related stimuli and by components of stress signal transduction pathways, such as the plant hormones jasmonic acid (JA), ethylene (Et) and salicylic acid (SA), as well as by pathogen infection has been demonstrated for a number of *ERF* genes [13]–[16].

The key roles of SA, JA and Et as signals mediating pathogen defense responses have been widely documented [17], [18]. Although exceptions have been reported, in general resistance to biotrophic and hemibiotrophic pathogens such as *Pseudomonas syringae* and *Hyaloperonospora parasitica* is mediated through SA-signalling, while resistance against necrotrophic pathogens such as *Botrytis cinerea* is mediated through JA/Et-signalling [18], [19]. It is apparent that extensive crosstalk exists between these two signalling pathways, with the majority of studies reporting a mutually antagonistic interaction [17], [20]. For example, application of exogenous SA suppresses the induction of JA-responsive genes such as *PDF1.2* [21], while the induction of SA-mediated defense responses in Arabidopsis following infection with *P. syringae* renders the plant more susceptible to infection by the necrotrophic pathogen *Alternaria brassicicola* by suppression of JA signalling [22]. Conversely, JA-signalling mutants such as *coi1* display elevated expression of the SA marker gene *PR-1* [23]. Plant pathogens have evolved mechanisms that exploit this mutual antagonism to subvert the host immune response. The phyototoxin coronatine produced by *P. syringae* is a jasmonoyl-isoleucine (JA-Ile) structural analogue and binds to the JA receptor COI1, resulting in the suppression of SA-mediated signalling [24], [25].

Despite the evidence that ERFs play important roles in many plant physiological processes, many of the 122 Arabidopsis ERFs have yet to be assigned a biological role. Of the group IX ERFs, the IXc subgroup has been the most studied and includes members such as ERF1, ERF14 and ORA59 with demonstrated roles in defense against microbial pathogens [26], [27]. In contrast, very little is known about the biological functions or downstream targets of members of the IXb subgroup.

We therefore investigated *ERF5* (At5g47230) and its closest relative in the IXb subgroup, *ERF6* (At4g17490), which shares 58% identity at the amino acid level [2]. To identify putative downstream targets of these transcription factors we carried out microarray analyses on transgenic Arabidopsis constitutively-expressing either *ERF5* or *ERF6*. These data suggested a redundant role for these two transcription factors as positive regulators of a subset of jasmonic acid/ethylene-responsive defense genes. Accordingly, a double *erf5 erf6* mutant displayed reduced expression of *PDF1.1* and *PDF1.2a* and increased susceptibility to the necrotrophic pathogen *B. cinerea*, while the single *erf5* and *erf6* mutants did not. Constitutive expression of either transcription factor resulted in enhanced resistance to *B. cinerea*, but increased susceptibility to avirulent *P. syringae*. Analysis of *PR-1* expression indicated that SA-mediated signalling may be repressed in these plants, providing further evidence for antagonism between JA/Et and SA-mediated signalling in plants.

## Results

### Plants constitutively expressing *ERF5* or *ERF6* display upregulation of pathogen defense genes

**Figure 1:**
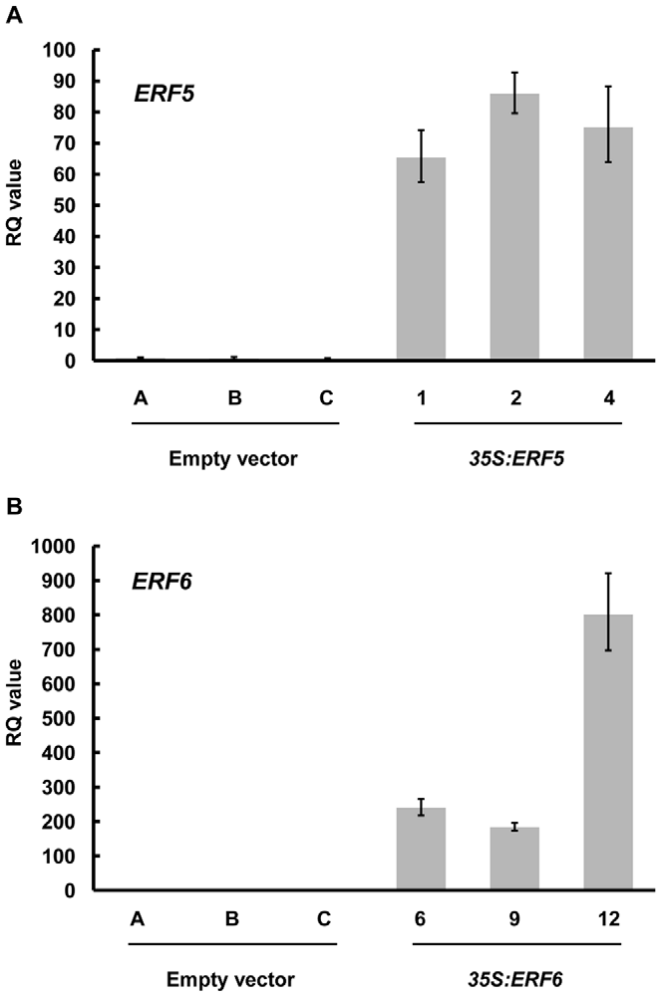
Analysis of transgene expression in *35S:ERF5* and *35S:ERF6* plants. Relative accumulation of (A) *ERF5* or (B) *ERF6* mRNA in ten-day old seedlings was measured by qRT-PCR in three constitutive-expressing lines (*35S:ERF5* lines 1, 2, 4 and *35S:ERF6* lines 6, 9, 12) and in three empty vector control lines (A, B and C). Relative Quantitation (RQ) values were calculated after normalization to *PEX4* expression levels. Each value is the mean of three technical replicates and the data are representative of three independent experiments. The RQ values of *ERF5* and *ERF6* in the empty vector lines are too low to be detected in the graph.

In order to identify potential downstream targets of ERF5 and ERF6, we generated transgenic plants constitutively expressing each of these transcription factors under the control of the CaMV *35S* promoter. RNA gel blot analysis was initially used to identify overexpressing lines (data not shown), and mRNA levels in the three highest overexpressors (*35S:ERF5* lines 1, 2 and 4; *35S AtERF6* lines 6, 9 and 12) were quantified by real-time PCR (Figure 1). These transgenic plants were subsequently used in expression profiling experiments to identify putative downstream targets of ERF5 and ERF6. Microarray analyses were performed as three biological repeats, using cDNA prepared from ten-day old seedlings for three independent transgenic lines of *35S:ERF5* and *35S:ERF6*.

In total, we identified 46 genes that showed significant (>2-fold) upregulation in the transgenic plants; 18 of these were upregulated in both *35S:ERF5* and *35S:ERF6* plants, while 15 were upregulated only in *35S:ERF5* plants, and 13 only in *35S:ERF6* plants (Table 1). Functional enrichment analysis of this set of 46 genes using FatiGO (http://babelomics.bioinfo.cipf.es) revealed a highly significant enrichment (Fisher exact test, two-tailed, *p* = 2.24^e-11^) for genes annotated with gene ontology (GO) term GO:0006952 (defense response), with 39.1% (18/46) of the upregulated genes associated with this term, compared to 2.61% in the whole Arabidopsis genome. Other significantly over-represented GO terms included response to fungus, response to bacterium and response to ethylene (Figure S1). Notably, 13 of the 18 (72.2%) genes significantly upregulated in both *35S:ERF5* and *35S:ERF6* plants were associated with the GO term defense response, including 6 plant defensin genes.

**Table 1:** Genes upregulated by constitutive expression of *ERF5* and *ERF6*.

AGI number	Gene annotation	*35S::ERF5*		*35S::ERF6*	
		Fold change	*p*-value	Fold change	*p*-value
*35S:ERF5* only					
At1g02030	Zinc finger (C2H2 type) family protein	3.7	0.002	-	-
At1g09415	NIM1-interacting protein 3 (NIMIN-3)	2.8	0.004	-	-
At1g33780	Unknown protein	2.6	0.007	-	-
**At2g02930**	**Glutathione ** ***S*** **-transferase (GST16)**	**2.8**	**0.006**	-	-
At2g41640	Glycosyltransferase	2.7	0.007	-	-
At3g04960	Unknown protein	3.0	0.008	-	-
**At3g16530**	**Lectin-like protein**	**6.6**	**<0.001**	-	-
At3g49620	Dark inducible 11 (DIN11)	3.3	0.002	-	-
**At3g53260**	**Phenylalanine ammonia-lyase 2 (PAL2)**	**6.4**	**<0.001**	-	-
At3g55850	Long after far-red 3 (LAF3)	4.1	0.001	-	-
At5g17960	DC1 domain-containing protein	3.6	0.006	-	-
At5g18980	Unknown protein	3.0	0.01	-	-
At5g39100	Germin-like protein 6 (GLP6)	3.7	0.002	-	-
At5g47230	Ethylene-responsive element binding factor 5 (ERF5)	24.6	0.001	-	-
At5g57785	Unknown protein	2.5	0.01	-	-
*35S:ERF5* and *35S:ERF6*					
**At1g02920**	**Glutathione ** ***S*** **-transferase (GST11)**	**6.7**	**<0.001**	**6.3**	**<0.001**
**At1g02930**	**Glutathione ** ***S*** **-transferase (GST1)**	**7.8**	**<0.001**	**5.1**	**<0.001**
**At1g55010**	**Plant defensin (PDF1.5)**	**24.4**	**<0.001**	**9.8**	**<0.001**
**At1g75830**	**Plant defensin (PDF1.1)**	**15.8**	**<0.001**	**21.5**	**<0.001**
At1g78850	Curculin-like (mannose-binding) lectin family protein	3.7	0.002	2.4	0.005
At1g78860	Curculin-like (mannose-binding) lectin family protein	4.1	0.002	2.7	0.003
At2g25735	Unknown protein	2.9	0.005	2.1	0.004
**At2g26010**	**Plant defensin (PDF1.3)**	**13.0**	**<0.001**	**11.1**	**<0.001**
**At2g26020**	**Plant defensin (PDF1.2b)**	**19.5**	**<0.001**	**18.2**	**<0.001**
**At2g26560**	**Patatin-like protein 2 (PLP2)**	**2.7**	**0.006**	**2.9**	**0.003**
**At3g04720**	**Pathogenesis-related 4 (PR-4)**	**3.6**	**0.004**	**3.5**	**<0.001**
**At3g15356**	**Legume lectin family protein**	**4.4**	**0.002**	**3.4**	**<0.001**
**At3g49110**	**Peroxidase (PRX33)**	**3.7**	**0.003**	**3.3**	**<0.001**
At4g06746	DREB and EAR motif protein 5 (DEAR5/RAP2.9)	3.0	0.007	2.9	0.002
**At4g16260**	**Glycoside hydrolase**	**4.1**	**0.001**	**3.5**	**<0.001**
At4g33720	Pathogenesis-related protein	25.2	<0.001	19.0	<0.001
**At5g44420**	**Plant defensin (PDF1.2a)**	**43.3**	**<0.001**	**27.4**	**<0.001**
**At5g44430**	**Plant defensin (PDF1.2c)**	**11.8**	**<0.001**	**11.8**	**<0.001**
*35S:ERF6* only					
At1g03905	ABC transporter family protein	-	-	3.7	<0.001
At1g21245	Wall-associated kinase-related	-	-	3.1	0.009
At1g27020	Unknown protein	-	-	3.0	<0.001
At1g49960	Xanthine/uracil permease family protein	-	-	2.0	0.008
**At1g53940**	**GDSL-motif lipase 2 (GLIP2)**	**-**	**-**	**2.5**	**0.009**
At2g18980	Peroxidase	-	-	3.8	<0.001
At3g45500	Unknown protein	-	-	3.0	0.001
At3g59930	Defensin-like family protein	-	-	2.7	0.004
**At4g11650**	**Osmotin-like protein (OSM34)**	**-**	**-**	**2.7**	**0.003**
At4g13450	Universal stress protein (USP) family protein	-	-	3.0	0.006
At4g17490	Ethylene-responsive element binding factor 6 (ERF6)	-	-	2.1	0.004
At4g17615	Calcineurin B-like protein 1 (CBL1)	-	-	3.1	0.001
At5g06390	Fasciclin-like arabinogalactan protein 17 (FLA17)	-	-	2.6	0.007

Fold change in transcript levels from plants constitutively expressing *ERF5* or *ERF6* compared to control plants transformed with the empty pK2GW7 vector. Fold change values are the average of the three independently transformed lines. All genes listed had a *p*-value of ≤0.01 and displayed expression ratios >2 in all three transgenic lines analysed. Genes in bold are annotated with the Gene Ontology term GO:0006952 (defense response). Gene annotations are from TAIR (www.arabidopsis.org).

**Figure 2:**
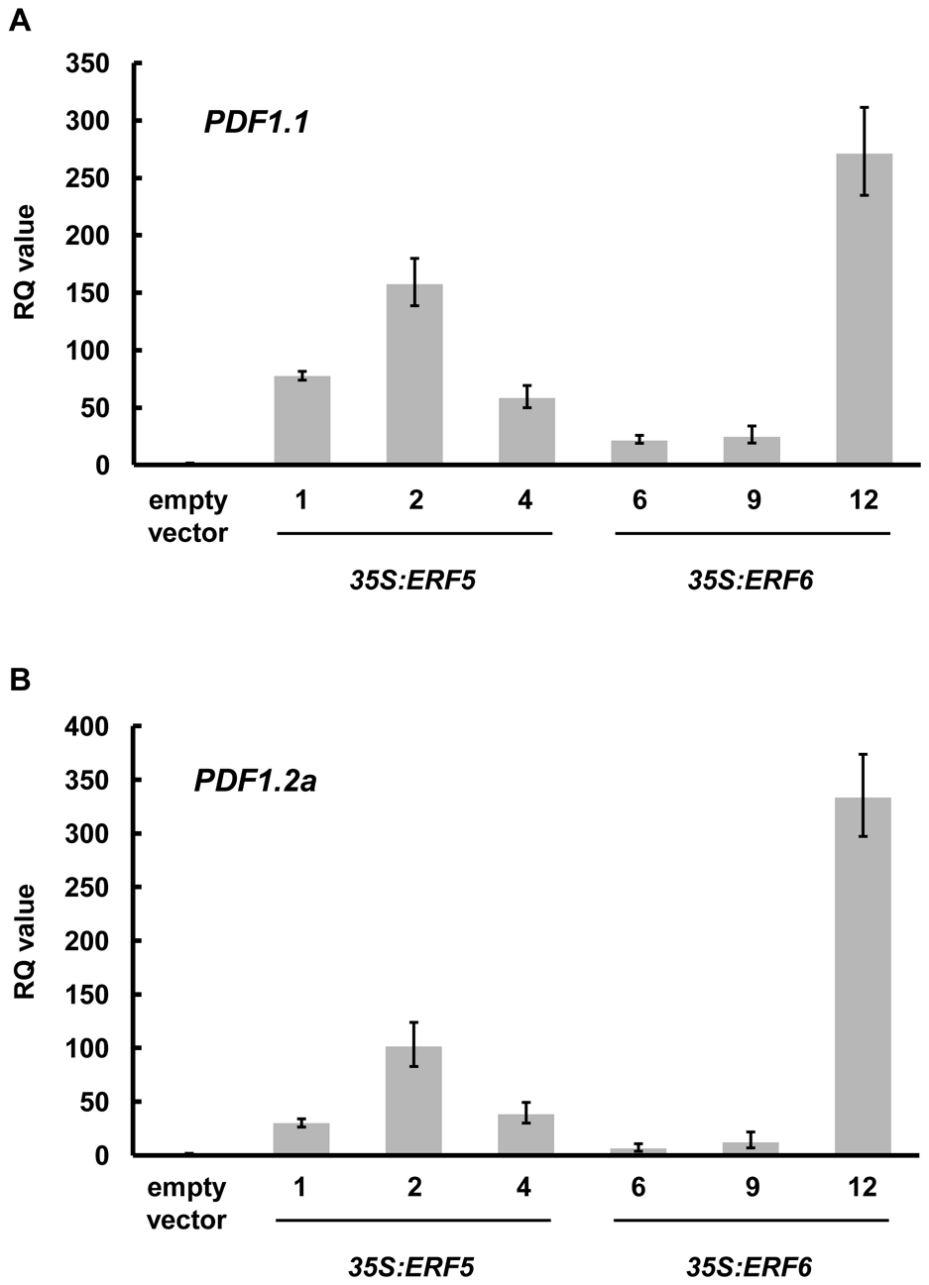
Validation of microarray data by qRT-PCR. Relative accumulation of (A) *PDF1.1* (At1g75830) and (B) *PDF1.2a* (At5g44420) mRNA in ten-day old seedlings of the three *35S:ERF5* and three *35S:ERF6* lines was measured by qRT-PCR. Relative Quantitation (RQ) values were calculated after normalization to *PEX4* expression levels. Each value is the mean of three technical replicates and the data are representative of three independent experiments. The RQ values of *ERF5* and *ERF6* in the empty vector lines are too low to be detected in the graph.

To confirm the validity of the microarray data, we performed real-time PCR analysis on *PDF1.1* (At1g75830) and *PDF1.2a* (At5g44420) expression levels. Both genes were, according to our microarray data, highly up-regulated in plants constitutively expressing *ERF5* or *ERF6*. As shown in Figure 2, the expression levels of both genes were higher in the *35S:ERF5* and *35S:ERF6* plants, as compared to the empty vector control, showing good agreement with the microarray data. Furthermore, the RQ values for both *PDF1.1* and *PDF1.2a* correlated with the level of *ERF* transgene expression in these plants for both *35S:ERF5* and *35S:ERF6* plants (Figures 1 and 2).

### Promoter analysis suggests that ERF5 and ERF6 play a role in JA/Et-mediated gene expression

The upstream promoter sequences of the 46 upregulated genes were analysed in order to identify over-represented oligonucleotide motifs that may represent transcription factor binding sites or regulatory sites. The GCC box (AGCCGCC) and GCC core (GCCGCC) were found to be significantly over-represented in the promoters of both the *35S:ERF5* and *35S:ERF6* upregulated genes (Tables S1 and S2). Furthermore, the observed/expected ratios for the GCC box were the highest of any 7-mer motif, at 13.9 in *35S:ERF5* and 14.2 in *35S:ERF6* plants respectively.

The GCC box can confer JA/Et-mediated regulation of promoter activity, and previous studies have identified a number of ERFs that can bind to this motif and either induce or repress gene expression [8], [22], [28]. Given the over-representation of the GCC box in the upstream regions of the *35S:ERF5*- and *35S:ERF6*-upregulated genes, we examined their JA/Et-responsiveness by comparison to microarray data previously generated by Pré et al. (2008). In this study, two-week old wild-type Arabidopsis plants were treated with either 50 µM JA or a combination of 50 µM JA and 1 mM ethephon (an ethylene releasing agent) for 8 or 24 h [27]. In total, 16 of the 46 genes upregulated in either *35S:ERF5* or *35S:ERF6* plants were also identified as JA/Et responsive by Pré and colleagues, including 12 of the 18 (80%) genes upregulated in both *35S:ERF5* and *35S:ERF6* plants (Table 2). These 16 genes are also upregulated by overexpression of *ORA59* [27], a member of the ERF IXc subgroup (Table 2). The over-representation of JA/Et-responsive genes in the lists of transcripts regulated by ERF5 and ERF6 is highly significant as determined by Chi-squared test (*p*<0.001).

**Table 2:** Genes upregulated in *35S:ERF5* or *35S:ERF6* plants that are responsive to jasmonic acid and ethylene treatment and overexpression of *ORA59*.

AGI number	Gene description	JA 8 h	JA 24 h	JA+E 8 h	JA+E 24 h	*ORA59* OE
At1g02920	Glutathione *S*-transferase (GST11)	-	4.2	3.8	-	3.5
At1g02930	Glutathione *S*-transferase (GST1)	-	4.8	4.0	-	4.5
At1g75830	Plant defensin (PDF1.1)	-	4.4	-	-	10.8
At2g02930	Glutathione *S*-transferase (GST16)^a^	-	-	-	-	4.5
At2g26010	Plant defensin (PDF1.3)	-	5.4	16.9	22.8	11.6
At2g26020	Plant defensin (PDF1.2b)	-	6.7	34.2	25.7	11.1
At2g26560	Patatin-like protein 2 (PLP2)	-	6.0	28.8	12.5	14.5
At3g04720	Pathogenesis-related 4 (PR-4)	-	-	3.5	5.1	5.0
At3g15356	Legume lectin family protein	-	5.5	11.8	10.2	21.2
At3g16530	Lectin-like protein^a^	-	5.1	9.1	7.8	16.6
At3g49620	Dark inducible 11 (DIN11)^a^	-	56.1	36.5	35.3	12.5
At4g06746	DREB and EAR motif protein 5	3.0	5.0	10.8	7.7	11.5
At4g11650	Osmotin-like protein (OSM34)^b^	-	-	3.4	8.2	8.5
At4g16260	Glycoside hydrolase	-	-	5.2	7.0	10.4
At5g44420	Plant defensin (PDF1.2a)	-	7.4	31.7	16.9	7.9
At5g44430	Plant defensin (PDF1.2c)	-	6.6	21.1	21.7	10.6

Fold change in transcript level observed in 14-d old wild-type Col-0 plants treated with 50 µM JA ±1 mM ethephon (E) for 8 or 24 h, or in plants overexpressing the ERF *ORA59* (data from Pré et al. 2008).

a upregulated in *35S:ERF5* plants only,

b upregulated in *35S:ERF6* plants only. All other genes are upregulated in both *35S:ERF5* and *35S:ERF6* plants.

### ERF5 and ERF6 play positive but redundant roles in defense against *Botrytis cinerea*

**Figure 3:**
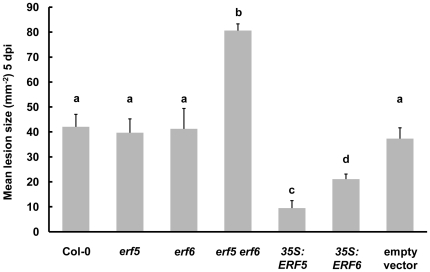
ERF5 and ERF6 play redundant roles as positive regulators of resistance against *Botrytis cinerea* in Arabidopsis. Detached leaves from four week-old plants were inoculated with *B. cinerea* spores, and lesion size (mm^−2^) measured after 5 days. ANOVA revealed a significant effect of host genotype (*p*<0.001) on lesion size 5 dpi. Mean lesion sizes with different letters are significantly different (*p*<0.05) as determined by Fisher LSD post-hoc analysis. Data shown are mean values +SD from three independent experiments. The *35S* lines analysed were *35S:ERF5* line 1 and *35S:ERF6* line 6.

Both the significant over-representation of defense-related and JA/Et-responsive genes in the 46 genes upregulated in the *35S:ERF5* and *35S:ERF6* plants, and the prevalence of the GCC box in their upstream sequences suggests that ERF5 and ERF6 may act as positive regulators of JA-mediated defense against necrotrophic pathogens. Accordingly, we found that constitutive expression of either transcription factor was sufficient to result in significantly increased resistance against the fungal necrotroph *B. cinerea* in comparison to that observed in wild-type plants or the empty vector control plants (Figure 3).

**Figure 4:**
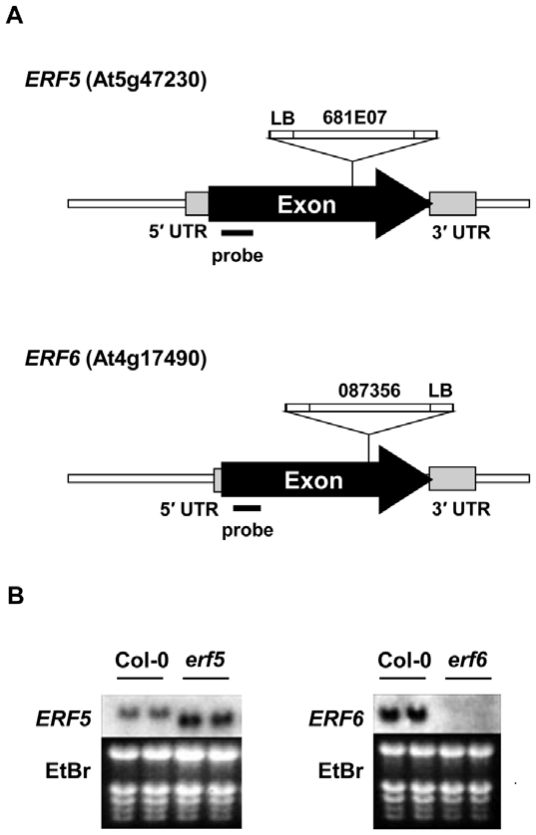
Analysis of *erf5* and *erf6* T-DNA insertion mutants. (A) Schematic representation of the *ERF5* and *ERF6* genes indicating the position of the T-DNA insertions. LB indicates the position of the left border of the T-DNA. (B) RNA gel blot analysis of *ERF5* and *ERF6* expression in the T-DNA mutants. Ten µg of total RNA was loaded per lane, equal loading is shown by ethidium bromide (EtBr) staining.

However, an enhanced disease resistance phenotype in an overexpressor line does not necessarily indicate that this gene performs a corresponding role in wild-type plants. In order to test whether ERF5 and ERF6 are indeed required for resistance against *B. cinerea* we isolated homozygous T-DNA insertion mutants (Figure 4A) from segregating populations (*erf5*: GABI_681E07 [29], *erf6*: SALK_087356 [30]) by PCR genotyping. RNA gel blot analysis of the homozygous lines revealed the production of an aberrant truncated *ERF5* transcript in the *erf5* mutant, while no *ERF6* transcript could be detected in the *erf6* mutant (Figure 4B). It is theoretically possible, though unlikely, that the truncated transcript of the *efr5* mutant has residual activity, thus this mutant might be a reduced, rather than loss of, function, mutant. The observations that 72% of the differentially expressed genes annotated with the GO term defense response (Table 1) and 12 of the 16 genes identified as JA/Et-responsive (Table 2) were upregulated in both *35S:ERF5* and *35S:ERF6* plants, suggests that any role played by ERF5 and ERF6 in JA-mediated defense against *B. cinerea* may be redundant. In order to test this, we generated a homozygous *erf5 erf6* double mutant, which showed greatly reduced expression of *ERF5* and *ERF6* as determined by qRT-PCR (Figure S2). While no significant difference in susceptibility to *B. cinerea* was observed in either the *erf5* or *erf6* mutants in comparison to wild-type plants, the *erf5 erf6* double mutant showed a significant increase in susceptibility to this pathogen (Figure 3). Together these data suggest that ERF5 and ERF6 play positive but redundant roles in defense against *B. cinerea* in Arabidopsis.

**Figure 5:**
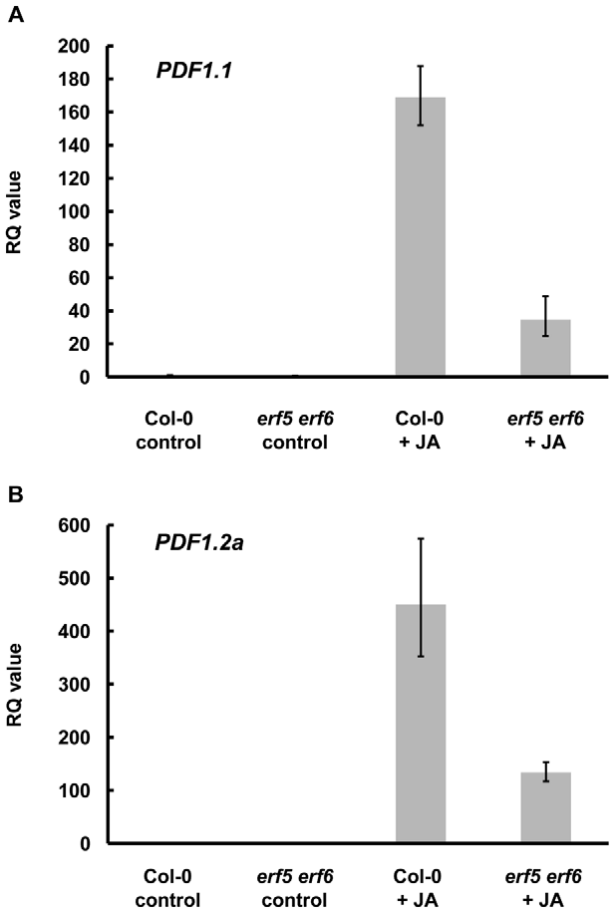
The *erf5 erf6* double mutant shows reduced JA-induction of plant defensin genes. Twelve-day old seedlings were treated with 100 µM MeJA (JA) or water (control) and harvested for RNA extraction after 24 h. Relative accumulation of (A) *PDF1.1* and (B) *PDF1.2a* mRNA was measured by qRT-PCR. Relative Quantitation (RQ) values were calculated after normalization to *PEX4* expression levels. Each value is the mean of three technical replicates and the data are representative of two independent experiments.

To determine whether the increased susceptibility of the *erf5 erf6* double mutant to *B. cinerea* might result from impairment of JA-mediated signalling, we analysed *PDF1.1* and *PDF1.2a* expression in these plants following treatment with 100 µM MeJA for 24 h. Expression levels of both of these genes were significantly lower in the *erf5 erf6* plants following JA treatment in comparison to those observed in wild-type plants (Figure 5), suggesting that JA-mediated gene expression is compromised in the double mutant.

### Constitutive expression of ERF5 or ERF6 reduces UV-C-induced SA-mediated *PR-1* expression and increases susceptibility to avirulent *Pseudomonas syringae*

**Figure 6:**
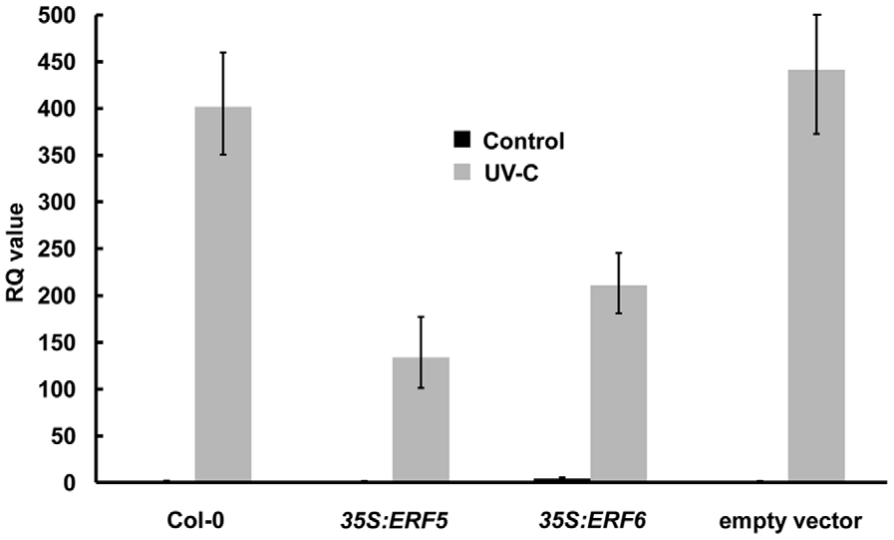
Constitutive expression of *ERF5* or *ERF6* reduces UVC-induced *PR-1* expression. Twelve-day old seedlings were irradiated with 5 kJ m^−2^ of UV-C and harvested for RNA extraction after 24 h. Relative accumulation of *PR-1* (At2g14610) mRNA was measured by qRT-PCR. Relative Quantitation (RQ) values were calculated for *PR-1* after normalization to At4g24410 expression levels. Each value is the mean of three technical replicates and the data are representative of two independent experiments. The *35S* lines analysed were *35S:ERF5* line 1 and *35S:ERF6* line 6.

**Figure 7:**
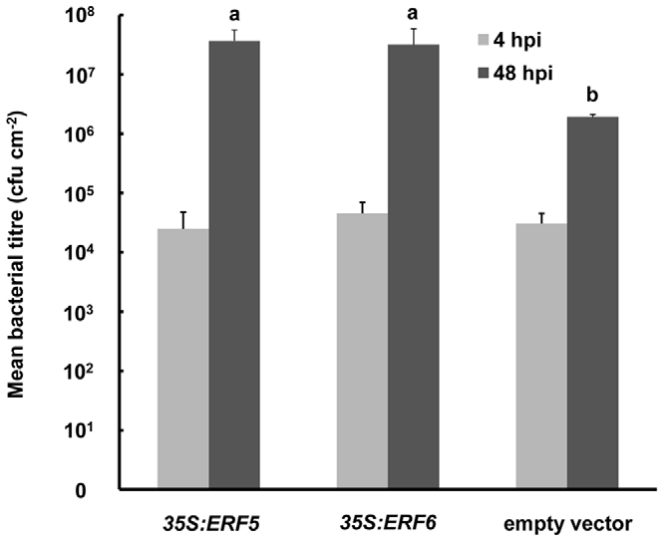
Constitutive expression of *ERF5* or *ERF6* leads to increased susceptibility to avirulent *Pseudomonas syringae*. Four-week old plants were infected with *Pst* DC3000 *avrB* (10^4^ cfu cm^−2^) and bacterial titres (cfu cm^−2^) determined at 4 and 48 hpi. ANOVA revealed a significant effect of host genotype (*p* = 0.004) on bacterial titre at 48 hpi. Mean bacterial titres with different letters are significantly different (*p*<0.05) as determined by Fisher LSD post-hoc analysis. Data shown are mean values +SD from three independent experiments. The *35S* lines analysed were *35S:ERF5* line 1 and *35S:ERF6* line 6.

The mutual antagonism between JA/Et and SA signalling is well known [18]. Given the constitutive upregulation of JA-responsive genes in the *35S:ERF5* and *35S:ERF6* plants we examined whether SA signalling was repressed in these plants by performing real-time PCR analysis on *PR-1*, a SA-inducible gene. Seedlings were exposed to UV-C, a treatment which has previously been shown to upregulate *PR-1* expression via SA signalling [31]. The *35S:ERF5* and *35S:ERF6* plants exhibited significantly reduced UV-C-induced *PR-1* expression in comparison to plants transformed with the empty vector (Figure 6). Since resistance against many biotrophic and hemibiotrophic pathogens is SA-dependent, we examined the response of the transgenic lines to an avirulent strain of the hemibiotroph bacterium *P. syringae* (*Pst*) DC3000 harbouring the *avrB* gene. Leaves of four-week old plants were infiltrated with a *Pst* DC3000 *avrB* suspension. Transgenic plants constitutively expressing either *ERF5* or *ERF6* were more susceptible to *Pst* DC3000 *avrB*, exhibiting significantly higher leaf bacterial titres 48 h post-infection in comparison to the empty vector control (Figure 7).

## Discussion

The production of plant defensins is a hallmark of the JA/Et-mediated defense response against necrotrophic pathogens [32], [33]. We identified a subset of defense genes, including six of the thirteen plant defensin genes in Arabidopsis, as putative downstream targets of ERF5 and ERF6 through expression profiling of plants constitutively expressing these transcription factors (Table 1, Figure 2). Analysis of the upstream regions of all of the putative target genes revealed an over-representation of the GCC-box (Tables S1 & S2). A number of ERFs have previously been shown to bind to this element within the promoters of JA/Et-responsive genes such as *PDF1.2a*, and either induce or repress gene expression [3], [4], [28]. While ERF5 and ERF6 might be acting indirectly on these motifs, the most parsimonious, and likely, explanation is that they also bind directly to these sequences, and function as GCC-box transcriptional activators. Indeed, a protoplast transactivation system has shown that ERF5 is able to activate the promoter of *PDF1.2*, providing support for a direct role in GCC-box promoter activation [34]. Constitutive expression of *ERF5* or *ERF6* thus leads to activation of JA/Et-dependent defense genes, and accordingly we found that *35S:ERF5* and *35S:ERF6* plants displayed increased resistance to the necrotrophic pathogen *Botrytis cinerea* (Figure 3).

Constitutive expression of several members of the ERF IXc subgroup such as *ORA59* (*ERF59*) and *ERF1* also results in increased expression of JA/Et-regulated defense genes including *PDF1.2a*, and in increased resistance to *B. cinerea* [26], [27]. Indeed, 16 of the 46 genes upregulated in the *35S:ERF5* and *35S:ERF6* plants are also upregulated in plants constitutively expressing *ORA59* (Table 2). However, these gain-of-function phenotypes are not necessarily indicative of a requirement for a given ERF in defense against *B. cinerea* in wild-type plants. For example, constitutive expression of an *ERF* gene may result in inappropriate binding to promoters that are not normally regulated by the transcription factor. Analysis of null mutants or RNAi lines is thus required to demonstrate that a given ERF is required for resistance to *B. cinerea*. While *ORA59*-silenced plants do indeed display increased susceptibility to *B. cinerea* [27], no such studies have been reported for *ERF1* to date. To determine whether ERF5 and ERF6 are required for resistance to *B. cinerea* in wild-type plants, we analysed the susceptibility of single *erf5* and *erf6* T-DNA insertion mutants, and a double *erf5 erf6* knockout to this pathogen. While neither of the single mutants displayed altered resistance to *B. cinerea*, the *erf5 erf6* double mutant showed a significant increase in susceptibility in comparison to wild-type plants (Figure 3). These data suggest that ERF5 and ERF6 play redundant roles in JA/Et-mediated defense against *B. cinerea* in Arabidopsis. This hypothesis is supported by the overlap in potential downstream targets of these two transcription factors (Table 1). Similar to *ORA59* and *ERF1*, the transcripts of *ERF5* and *ERF6* increase in abundance in response to treatment with either JA or Et, although fold induction is less (Table S3). However, unlike *ORA59* and *ERF1*, the transcripts of *ERF5* and *ERF6* do not increase in response to *Botrytis* infection (Table S4).

Notably, the *erf5 erf6* double mutant displayed reduced induction of *PDF1.1* and *PDF1.2a* expression in response to JA treatment (Figure 5), suggesting that the increased susceptibility of the mutant to *B. cinerea* may be explained in part by the abrogation of JA-mediated gene expression. The redundant roles of ERF5 and ERF6 in defense against *B. cinerea* are in contrast to that of ORA59; *ORA59*-silenced plants are not able to induce *PDF1.2a* in response to JA, and show increased susceptibility to *B. cinerea* infection [27]. Similarly, ERF14 plays a non-redundant role against *Fusarium oxysporum* [9]. In contrast to the severe growth retardation that was reported for constitutive expression of *ERF1*, *ERF14* and *ORA59* [9], [26], [27] *35S:ERF6* plants displayed no visible phenotype under normal growth conditions, while *35S:ERF5* plants were only slightly smaller than wild-type plants (data not shown). No difference in time to flowering was observed, and both lines produced viable seed.

ERF5 and ERF6 have recently been shown to interact *in planta*, and have been proposed to form part of a signalling network activated following the perception of the fungal PAMP chitin [35]. Plants constitutively expressing *ERF5* displayed increased susceptibility to the fungal necrotroph *Alternaria brassicicola* while a *erf5 erf6* double mutant displayed a modest reduction in spore production, but exhibited no apparent difference in lesion size in comparison to wild-type plants [35]. These results are in apparent contradiction to our results, where the *35S:ERF5* and *35S:ERF6* plants displayed increased resistance, and the *erf5 erf6* double mutant increased susceptibility to *B. cinerea*. This discrepancy might be attributable to the fact that these two plant-pathogen interactions differ somewhat. Wild-type Arabidopsis plants are resistant to *A. brassicicola*, developing small necrotic lesions that do not spread beyond the initial inoculation droplet [36], and the interaction is thus incompatible. In contrast, spreading necrotic lesions are observed during the compatible Arabidopsis-*B. cinerea* interaction. There are undoubtedly commonalities in the defense mechanisms employed against the necrotrophs e.g. JA levels increase in Arabidopsis following infection with either pathogen, and the JA-insensitive *coi1* mutant displays increased susceptibility to both pathogens [37]. However, a recent hierarchical cluster analysis of the expression profiles induced in Arabidopsis 24 h after infection by different plant pathogens revealed an unexpected and distinct lack of similarity between the profiles observed in response to *B. cinerea* and *A. brassicicola* [38]. Notably, several clusters of genes up-regulated in response to *B. cinerea* were down-regulated by *A. brassicicola*, and vice versa. Clearly then the host response to these pathogens is not identical, and it is thus possible that a given protein could play opposing roles against these two pathogens. Interestingly the ERF ORA59 also plays differential roles in defense against these pathogens; while ORA59 is required for *PDF1.2a* expression following infection with both pathogens, *ORA59*-silenced plants showed increased susceptibility only to *B. cinerea* and not to *A. brassicicola* [27].

The mutual antagonism between the JA/Et and SA signalling pathways is well-established [17], [20], and allows plants to mount an appropriate defense response against the attacking pathogen. Given that the JA/Et pathway was up-regulated in plants constitutively expressing *ERF5* or *ERF6*, we tested whether SA-mediated signalling was repressed. Consistent with a suppression of SA signalling, UV-C–induced *PR-1* expression was significantly reduced in *35S:ERF5* and *35S:ERF6* plants (Figure 6). Plants constitutively expressing *ERF5* or *ERF6* also showed increased susceptibility to the hemibiotroph *Pst* DC3000 *avrB* in comparison to empty vector control plants (Figure 7). These data suggest that ERF transcription factors can also play a role in the suppression of SA-mediated signalling, in addition to their previously reported role in the activation of JA/Et mediated responses. Plants constitutively expressing *ERF1* also show increased susceptibility to virulent *Pst* DC3000 [26]. While the molecular basis of this phenotype was not investigated, it is conceivable that *ERF1* overexpression also results in the suppression of SA-mediated defense responses. Further evidence that ERF transcription factors influence SA-mediated signalling comes from a recent report suggesting that ERF9 (group VIII) and ERF14 (group IX) suppress expression of *PR-1* during colonization of the host by the endophytic fungus *Piriformospora indica* [39].

The data presented here demonstrate a redundant role for ERF5 and ERF6 in defense against the necrotrophic pathogen *B. cinerea*. We suggest that these transcription factors function in the activation of JA/Et-responsive gene expression, and perhaps also in the suppression of SA-mediated signalling to optimize the host response against necrotrophic pathogens. Whether other members of the ERF IXb subgroup play a similar role remains to be determined.

## Materials and Methods

### Plant growth conditions

*Arabidopsis thaliana* plants were grown on 1× Murashige and Skoog (MS) 0.8% (w/v) agar plates or on peat (Jiffy Products, International AS, Norway) and vermiculite in a 1∶1 (v/v) ratio. Lighting was maintained at 150 µmol m^−2^ s^−1^ with a 16/8 h photoperiod and a temperature of 20°C.

### Generation of *35S:ERF5* and *35S:ERF6* lines

Full-length *ERF5* or *ERF6* cDNAs were cloned into the pK2GW7 vector which contains the cauliflower mosaic virus *35S* promoter [40]. Control plants were transformed with the empty pK2GW7 vector. *Agrobacterium*-mediated floral dip transformation of Col-0 plants was performed using the *Agrobacterium tumefaciens* strain C58C1, as described previously [41]. Transformants were selected on the basis of their ability to grow on MS medium containing 50 µg mL^−1^ kanamycin.

### Identification of *erf5* and *erf6* mutants and generation of double mutant

Segregating T-DNA insertion mutants (*erf5*: GABI_681E07 [29], *erf6*: SALK_087356 [30]) were obtained from the Nottingham Arabidopsis Stock Centre and homozygous lines were isolated by PCR genotyping. For PCR screening, genomic DNA was extracted from the unopened flower buds of individual plants and the following gene specific primers used in conjunction with the appropriate left border (LB) primer for screening (*ERF5* L: GGAATTTCTATCGATTCCATTTGA; *ERF5* R: GAACAACTTCACATAACGCC; GABI LB: ATATTGACCATCATACTCATTGC; *ERF6* L: CGACAAAGAAGCGTTTAGAC; *ERF6* R: GTGTTATGTGTTCTCTGTTC; SALK LB: TGGTTCACGTAGTGGGCCATCG). Homozygous *erf5* and *erf6* mutants were crossed, and homozygous *erf5 erf6* double mutants identified by PCR genotyping using the primers listed above.

### RNA blot analysis

RNA was isolated from whole seedlings by using the RNeasy Plant Mini Kit (Qiagen) according to the manufacturer's instructions. For RNA blot analysis, 10 µg of total RNA extracted from 10-day old seedlings was loaded on a 1% (v/v) agarose formaldehyde denaturing gel, transferred onto a nylon membrane, hybridized and washed as described previously [42]. The blots were hybridized with either an *ERF5* probe (PCR amplified with the primers CATCGAGAAACATCTACTCG and GTTTAGTAACTTCCGGTTTG) or *ERF6* probe (amplified using GTCTCCGTTGCCTACTACTG and CGATTGGATTGAACAGTAAC).

### Real-time quantitative PCR

Gene expression levels were analysed by quantitative real-time PCR using an Applied Biosystems 7300 system. A High Capacity cDNA reverse transcription kit (Applied Biosystems) was used to reverse transcribe cDNA from 2 µg of total RNA extracted using the RNeasy Plant Mini Kit (Qiagen) in conjunction with RNAse-free DNase (Qiagen) to remove any genomic DNA contamination. Quantitative real-time PCR (qRt-PCR) was used to detect relative transcript levels using either gene-specific TaqMan probes or gene-specific primers with SYBR green. Gene-specific primer pairs were designed using Primer Express software (Applied Biosystems) for *ERF5* (At5g47230), *ERF6* (At4g17490), *PDF1.1* (At1g75830) and *PDF1.2a* (At5g44420). Primers were *ERF5* forward TCTTCGGATCATCGTCCTCTTC; *ERF5* reverse GGTTTGCATACGGATTCAGAGAA; *ERF6* forward GAAAACCGCCGTTGAAGATC; *ERF6* reverse CGGTTGCGAATTGAATCCA; *PDF1.1* forward taaacaatagtcATGGCTAAGTCTGC; *PDF1.1* reverse ACTTGGCCTCTCGCACAACT; *PDF1.2a* forward AATCTTTGGTGCTAAATCGTGTGTAT; *PDF1.2a* reverse CAACGGGAAAATAAACATTAAAACAG). Expression levels were normalized to the expression of *PEX4* (At5g25760), an endogenous control gene used previously [40] (primers were forward: TCATAGCATTGATGGCTCATCCT and reverse: ACCCTCTCACATCACCAGATCTTAG). Five µL of a 1∶50 dilution of cDNA was amplified in a 15 µL reaction with Roche Faststart Universal SYBR Green Mastermix (ROX) (Roche) in an optical 96-well plate with three technical replicates for each sample. *PR-1* (At2g14610) transcripts were detected using a gene-specific TaqMan probe (Applied Biosystems probe identifier At02170748_s1) and expression levels were normalized to the expression of an endogenous control gene. We discovered that several commonly used endogenous control genes were strongly induced by UV-C irradiation (data not shown), therefore, in these experiments, we normalized to the expression of At4g24410 (probe identifier At02239002_g1), a gene whose expression does not alter under such conditions (Genevestigator; https://www.genevestigator.com). For qRT-PCR reactions using Taqman probes, 6 µL of a 1∶50 dilution of cDNA was amplified in a 15 µL reaction with TaqMan Universal PCR Mix (Applied Biosystems) in an optical 96-well plate with three technical replicates for each sample. In all cases, relative quantitation was performed by the ΔΔC_T_ (comparative C_T_) method [43]. Relative Quantitation (RQ) values and estimates of statistical variation (SV) for each sample were calculated as previously [44]. The algorithm used is described in Relative Quantitation (RQ) algorithms, Applied Biosystems Real-Time PCR Systems Software, July 2007. Error bars represent RQ_MIN_ and RQ _MAX_ and constitute the acceptable error level for a 95% confidence level according to Student's *t*-test.

### Microarray analysis

Microarray experiments were conducted using Arabidopsis 70-mer oligonucleotide microarrays printed with the Operon Arabidopsis version 3.0 AROS oligo set (University of Arizona; http://www.arizona.edu/microarray/). Experiments were performed as three biological repeats using cDNAs prepared independently from three individual lines. Total RNA was extracted from 10-day old plants using the RNeasy Plant Mini kit (Qiagen) and quantified using a Nanodrop ND-1000 spectrophotometer (Labtech). Integrity was checked using a 2100 Bioanalyzer and RNA Nano Chips (Agilent), according to manufacturer's instructions.

Reagents and enzymes for the preparation of materials for microarray hybridizations were sourced from the 3DNA 900 indirect labelling kit (Genisphere) unless otherwise stated. Two micrograms of total RNA was reverse-transcribed into unlabelled cDNA using SuperScript III reverse transcriptase (Invitrogen). Microarray slides were baked at 80°C for 30 min and then UV cross-linked at 300 MJ. Slides were then pre-hybridized in 3.5× SSC, 0.1% (w/v) SDS and 10 mg mL^−1^ bovine serum albumin (BSA) at 65°C for 20 min. Following pre-hybridization, slides were washed with distilled water, then isopropanol, dried with an airbrush and pre-scanned to check for any array defects. The capture sequence-tagged cDNAs were hybridized onto the microarray slide for 16 h at 55°C in a SlideBooster SB400 (Advalytix) with the power setting at 27 and a pulse∶pause ratio of 3∶7. Following the first hybridization, the slides were washed in 2× SSC, 0.2% (w/v) SDS for 10 min at 55°C, followed by washes with 2× SSC and 0.2× SSC for 10 min, at room temperature. The slides were dried with an airbrush and hybridized with the Cy3 and Cy5 3DNA dendrimer capture reagents (Genisphere) at 55°C for 4 h, and washed as before. Dried slides were scanned using a ScanArray Express HT (Perkin Elmer) using autocalibration to obtain optimized non-saturating images for each fluorophore.

Scanned microarray images were straightened, if necessary, with ImageViewer (BlueGnome; http://www.cambridgebluegnome.com/) and analysed using BlueFuse for Microarrays (BlueGnome). Spot data were extracted from images and manually flagged to remove hybridization artefacts before fusion. Fused data were filtered according to the pON value. Spots with pON values <0.5 in both channels were excluded to eliminate the bias generated by the inclusion of unhybridized spots in the statistical interpretation of the data, and the data were globally adjusted such that the mean rRNA ratio was 1.0. The data were then analysed using a locally prepared implementation of the Cyber-T algorithm within BASE [45] maintained by the Computational Biology Research Group at the University of Oxford as described previously [46]. A cut off *p*-value of 0.01 was used to identify differentially expressed genes. Genes whose transcript levels did not change consistently (*i.e.* with an expression ratio greater than or less than one in all three replicate experiments) were discarded. Total microarray data have been deposited in the ArrayExpress database (www.ebi.ac.uk/arrayexpress) under the accession numbers E-MTAB-436 (*35S:ERF5*) and E-MTAB-435 (*35S:ERF6*) (www.ebi.ac.uk/aerep/login; username: Reviewer_E-MTAB-436, password 1289219822065 and username: Reviewer_E-MTAB-435, password: 1289228646825).

### Promoter motif analysis

Promoter sequences (1000 bp upstream) were downloaded from the TAIR database (http://www.arabidopsis.org/tools/bulk/sequences/index.jsp), and analysed for over-represented promoter motifs using the “oligo-analysis” tool (default settings, Markov chain order 2) available online at the Regulatory Sequence Analysis Tools (RSAT) website (http://rsat.ulb.ac.be/rsat) [47]. Sequences were searched for oligomers between 4 and 8 bp in length. Only motifs with a *p*-value <0.001 were considered significant. All over-represented motifs were then compared to those listed in the PLACE database of plant *cis*-acting regulatory DNA elements (http://www.dna.affrc.go.jp/PLACE/) to determine whether they had been previously characterized.

### Pathogen assays

*Botrytis cinerea* (pepper isolate) was maintained on apricot halves at 22°C, and spores collected in 3 mL water 12 days after initial inoculation. Spore number was determined using a haemocytometer and adjusted to 5,000 spores mL^−1^ in 50% (v/v) grape juice. Single leaves were excised from ten four-week old plants per plant line and placed on 1% (w/v) agar on large petri dishes. Leaves were inoculated with 10 µL of the spore suspension, and the plates were sealed with parafilm to maintain humidity. Photographs were taken five days after inoculation, and the area of the necrotic lesion determined using ImageJ software (http://rsbweb.nih.gov/ij/).

Avirulent *Pseudomonas syringae* pv. *tomato* (*Pst*) DC3000 carrying the *AvrB* gene was grown in King's broth (KB) supplemented with 50 µg mL^−1^ rifampicin and 10 µg mL^−1^ tetracycline. Four-week old plants were infected with a *Pst* suspension at an OD_600 nm_ of 0.002 (corresponding to 10^4^ colony forming units cm^2^) in 10 mM MgCl_2_ by infiltration of the leaf using a needleless 1 mL syringe. Three leaves were harvested per plant from a total of three plants at 4 h post-infection (hpi) and from a further three plants at 48 hpi. Single leaf discs of 0.5 cm^2^ were obtained from each leaf sample and pooled per plant, giving three biological replicates per time point. The disks were ground in 1 mL 10 mM MgCl_2_ and serial dilutions made from the resulting suspensions. Ten µL of each dilution was spotted onto KB agar plates containing 50 µg mL^−1^ rifampicin, and colonies were counted after 2 d growth at 30°C.

ANOVA was used to determine whether host genotype had a significant effect on susceptibility to *B. cinerea* or *P. syringae*, followed by Fisher LSD post-hoc analysis to identify mean values significantly different at *p* = 0.05. Prior to ANOVA, Raw data were transformed, using square root transformation for lesion sizes and natural logs for bacterial titres to ensure homogeneity of variance and normality of error.

### JA treatment

Seeds were sown individually and evenly on horizontal 1× MS agar plates. After 12 days seedlings were transferred to water and left overnight. The following day, methyl jasmonic acid was added to a final concentration of 100 µM, and seedlings harvested after 24 h for RNA extraction.

### UV treatment

Seeds were sown individually and evenly on horizontal 1× MS agar plates. After 12 days lids were removed from the plates and the seedlings were irradiated with 5 kJ m^−2^ of UV-C, (wavelength 254 nm) in a UV cross-linker (Uvitec). Immediately after irradiation all plates, including control plates, were resealed with micropore tape and returned to the growth chamber. After 24 h samples were harvested for RNA extraction.

## Supporting Information

Figure S1 **Significantly over-represented GO terms in the genes upregulated in** ***35S:ERF5*** **or** ***35S:ERF6*** **plants.** Directed acyclic hierarchical graph (DAG) of significantly over-represented gene ontology (GO) terms in the genes upregulated in *35S:ERF5* or *35S:ERF6* plants. The DAG was generated using FatiGO (http://babelomics.bioinfo.cipf.es). GO terms in red are significantly over-represented in the dataset.
(PDF)

Figure S2 **The** ***erf5 erf6*** **double mutant shows reduced expression of** ***ERF5*** **and** ***ERF6*** **.** Relative accumulation of *ERF5* or *ERF6* mRNA was measured by qRT-PCR in ten-day old seedlings. Relative Quantitation (RQ) values were calculated after normalization to *PEX4* expression levels. Each value is the mean of three technical replicates and the data are representative of three independent experiments.
(PDF)

Table S1 **Promoter motif enrichment analysis of genes significantly upregulated in** ***35S:ERF5*** **plants.**
(PDF)

Table S2 **Promoter motif enrichment analysis of genes significantly upregulated in** ***35S:ERF6*** **plants.**
(PDF)

Table S3 **Fold induction values of** ***ERF5*** **,** ***ERF6*** **,** ***ERF1*** **and** ***ORA59*** **in response to ethylene and jasmonic acid treatment.** Fold change in transcript level observed in 7-d old wild-type Col-0 plants treated with 10 µM ACC or 10 µM MeJA for 0.5, 1 or 3 h. Microarray data from the AtGenExpress project with the TAIR submission number ME00334 (ACC) and ME00337 (MeJA) [48]. Values obtained from the eFP Browser on the Botany Array Resource (BAR) [49].
(PDF)

Table S4 **Fold induction values of** ***ERF5*** **,** ***ERF6*** **,** ***ERF1*** **and** ***ORA59*** **in response to** ***Botrytis cinerea*** **infection.** Fold change in transcript level observed in 4-week old wild-type Col-0 plants inoculated with *B. cinerea* spores at 18 or 48 h post-inoculation. Microarray data from the AtGenExpress project with the TAIR submission number ME00341. Values obtained from the eFP Browser on the Botany Array Resource (BAR) [49].
(PDF)

## References

[1] Okamuro JK, Caster B, Villarroel R, Van Montagu M, Jofuku KD (1997). The AP2 domain of APETALA2 defines a large new family of DNA binding proteins in Arabidopsis.. Proceedings of the National Academy of Sciences of the United States of America.

[2] Nakano T, Suzuki K, Fujimura T, Shinshi H (2006). Genome-wide analysis of the ERF gene family in Arabidopsis and rice.. Plant Physiology.

[3] Ohme-Takagi M, Shinshi H (1995). Ethylene-Inducible DNA-Binding Proteins That Interact with an Ethylene-Responsive Element.. Plant Cell.

[4] Fujimoto SY, Ohta M, Usui A, Shinshi H, Ohme-Takagi M (2000). Arabidopsis ethylene-responsive element binding factors act as transcriptional activators or repressors of GCC box-mediated gene expression.. Plant Cell.

[5] Song CP, Agarwal M, Ohta M, Guo Y, Halfter U (2005). Role of an Arabidopsis AP2/EREBP-type transcriptional repressor in abscisic acid and drought stress responses.. Plant Cell.

[6] Berrocal-Lobo M, Molina A (2004). Ethylene Response Factor 1 mediates Arabidopsis resistance to the soilborne fungus *Fusarium oxysporum*.. Mol Plant Microbe Interact.

[7] Solano R, Stepanova A, Chao Q, Ecker JR (1998). Nuclear events in ethylene signaling: a transcriptional cascade mediated by ETHYLENE-INSENSITIVE3 and ETHYLENE-RESPONSE-FACTOR1.. Genes Dev.

[8] McGrath KC, Dombrecht B, Manners JM, Schenk PM, Edgar CI (2005). Repressor- and activator-type ethylene response factors functioning in jasmonate signaling and disease resistance identified via a genome-wide screen of Arabidopsis transcription factor gene expression.. Plant Physiology.

[9] Oñate-Sánchez L, Anderson JP, Young J, Singh KB (2007). AtERF14, a member of the ERF family of transcription factors, plays a nonredundant role in plant defense.. Plant Physiology.

[10] van der Graaff E, Den Dulk-Ras A, Hooykaas PJJ, Keller B (2000). Activation tagging of the *LEAFY PETIOLE* gene affects leaf petiole development in *Arabidopsis thaliana*.. Development.

[11] Rashotte AM, Mason MG, Hutchison CE, Ferreira FJ, Schaller GE (2006). A subset of Arabidopsis AP2 transcription factors mediates cytokinin responses in concert with a two-component pathway.. Proceedings of the National Academy of Sciences of the United States of America.

[12] Wilson K, Long D, Swinburne J, Coupland G (1996). A dissociation insertion causes a semidominant mutation that increases expression of *TINY*, an Arabidopsis gene related to *APETALA2*.. Plant Cell.

[13] Brown RL, Kazan K, McGrath KC, Maclean DJ, Manners JM (2003). A role for the GCC-box in jasmonate-mediated activation of the *PDF1.2* gene of Arabidopsis.. Plant Physiology.

[14] Chen WQ, Provart NJ, Glazebrook J, Katagiri F, Chang HS (2002). Expression profile matrix of Arabidopsis transcription factor genes suggests their putative functions in response to environmental stresses.. Plant Cell.

[15] Cheong YH, Chang HS, Gupta R, Wang X, Zhu T (2002). Transcriptional profiling reveals novel interactions between wounding, pathogen, abiotic stress, and hormonal responses in Arabidopsis.. Plant Physiology.

[16] Gu YQ, Yang C, Thara VK, Zhou J, Martin GB (2000). *Pti4* is induced by ethylene and salicylic acid, and its product is phosphorylated by the Pto kinase.. Plant Cell.

[17] Verhage A, van Wees SC, Pieterse CM (2010). Plant immunity: it's the hormones talking, but what do they say?. Plant Physiol.

[18] Grant MR, Jones JD (2009). Hormone (dis)harmony moulds plant health and disease.. Science.

[19] Glazebrook J (2005). Contrasting mechanisms of defense against biotrophic and necrotrophic pathogens.. Annual Review of Phytopathology.

[20] Koornneef A, Pieterse CM (2008). Cross talk in defense signaling.. Plant Physiol.

[21] Koornneef A, Leon-Reyes A, Ritsema T, Verhage A, Den Otter FC (2008). Kinetics of salicylate-mediated suppression of jasmonate signaling reveal a role for redox modulation.. Plant Physiol.

[22] Spoel SH, Johnson JS, Dong X (2007). Regulation of tradeoffs between plant defenses against pathogens with different lifestyles.. Proc Natl Acad Sci U S A.

[23] Kazan K, Manners JM (2008). Jasmonate signaling: toward an integrated view.. Plant Physiol.

[24] Brooks DM, Bender CL, Kunkel BN (2005). The *Pseudomonas syringae* phytotoxin coronatine promotes virulence by overcoming salicylic acid-dependent defences in *Arabidopsis thaliana*.. Mol Plant Pathol.

[25] Katsir L, Schilmiller AL, Staswick PE, He SY, Howe GA (2008). COI1 is a critical component of a receptor for jasmonate and the bacterial virulence factor coronatine.. Proc Natl Acad Sci U S A.

[26] Berrocal-Lobo M, Molina A, Solano R (2002). Constitutive expression of *ETHYLENE-RESPONSE-FACTOR1* in Arabidopsis confers resistance to several necrotrophic fungi.. Plant Journal.

[27] Pré M, Atallah M, Champion A, De Vos M, Pieterse CM (2008). The AP2/ERF domain transcription factor ORA59 integrates jasmonic acid and ethylene signals in plant defense.. Plant Physiol.

[28] Zarei A, Korbes AP, Younessi P, Montiel G, Champion A (2011). Two GCC boxes and AP2/ERF-domain transcription factor ORA59 in jasmonate/ethylene-mediated activation of the PDF1.2 promoter in Arabidopsis.. Plant molecular biology.

[29] Kleinboelting N, Huep G, Kloetgen A, Viehoever P, Weisshaar B (2012). GABI-Kat SimpleSearch: new features of the Arabidopsis thaliana T-DNA mutant database.. Nucleic Acids Res.

[30] Alonso JM, Stepanova AN, Leisse TJ, Kim CJ, Chen H (2003). Genome-wide insertional mutagenesis of Arabidopsis thaliana.. Science.

[31] Nawrath C, Heck S, Parinthawong N, Métraux JP (2002). EDS5, an essential component of salicylic acid-dependent signaling for disease resistance in Arabidopsis, is a member of the MATE transporter family.. Plant Cell.

[32] Penninckx IA, Thomma BP, Buchala A, Metraux JP, Broekaert WF (1998). Concomitant activation of jasmonate and ethylene response pathways is required for induction of a plant defensin gene in Arabidopsis.. The Plant cell.

[33] Laluk KaM T (2010). Necrotroph attacks on plants: Wanton destruction or covert extortion?.

[34] Wehner N, Hartmann L, Ehlert A, Bottner S, Onate-Sanchez L (2011). High-throughput protoplast transactivation (PTA) system for the analysis of Arabidopsis transcription factor function.. Plant J.

[35] Son GH, Wan J, Kim HJ, Nguyen XC, Chung WS (2012). Ethylene-Responsive Element-Binding Factor 5, ERF5, Is Involved in Chitin-Induced Innate Immunity Response.. Molecular plant-microbe interactions: MPMI.

[36] van Wees SCM, Chang HS, Zhu T, Glazebrook J (2003). Characterization of the early response of Arabidopsis to Alternaria brassicicola infection using expression profiling.. Plant Physiology.

[37] Thomma BP, Eggermont K, Penninckx IA, Mauch-Mani B, Vogelsang R (1998). Separate jasmonate-dependent and salicylate-dependent defense-response pathways in Arabidopsis are essential for resistance to distinct microbial pathogens.. Proceedings of the National Academy of Sciences of the United States of America.

[38] Mulema JM, Denby KJ (2011). Spatial and temporal transcriptomic analysis of the Arabidopsis thaliana-Botrytis cinerea interaction..

[39] Camehl I, Sherameti I, Venus Y, Bethke G, Varma A (2010). Ethylene signalling and ethylene-targeted transcription factors are required to balance beneficial and nonbeneficial traits in the symbiosis between the endophytic fungus Piriformospora indica and Arabidopsis thaliana.. New Phytologist.

[40] Karimi M, Inzé D, Depicker A (2002). GATEWAY™ vectors for *Agrobacterium*-mediated plant transformation.. Trends in Plant Science.

[41] Clough SJ, Bent AF (1998). Floral dip: a simplified method for *Agrobacterium*-mediated transformation of *Arabidopsis thaliana*.. Plant J.

[42] Ülker B, Peiter E, Dixon DP, Moffat C, Capper R (2008). Getting the most out of publicly available T-DNA insertion lines.. Plant Journal.

[43] Livak KJ, Schmittgen TD (2001). Analysis of relative gene expression data using real-time quantitative PCR and the 2^−ΔΔCT^ method.. Methods.

[44] Knight H, Mugford SG, Ülker B, Gao DH, Thorlby G (2009). Identification of SFR6, a key component in cold acclimation acting post-translationally on CBF function.. Plant Journal.

[45] Saal LH, Troein C, Vallon-Christersson J, Gruvberger S, Borg Å (2002). BioArray Software Environment (BASE): a platform for comprehensive management and analysis of microarray data.. Genome Biology.

[46] Okamoto H, Gobel C, Capper RG, Saunders N, Feussner I (2009). The alpha-subunit of the heterotrimeric G-protein affects jasmonate responses in Arabidopsis thaliana.. Journal of Experimental Botany.

[47] van Helden J (2003). Regulatory sequence analysis tools.. Nucleic Acids Res.

[48] Goda H, Sasaki E, Akiyama K, Maruyama-Nakashita A, Nakabayashi K (2008). The AtGenExpress hormone and chemical treatment data set: experimental design, data evaluation, model data analysis and data access.. Plant J.

[49] Winter D, Vinegar B, Nahal H, Ammar R, Wilson GV (2007). An “Electronic Fluorescent Pictograph” Browser for Exploring and Analyzing Large-Scale Biological Data Sets.. Plos One.

