# Supplementary material for: ERF5 and ERF6 Play Redundant Roles as Positive Regulators of JA/Et-Mediated Defense against Botrytis cinerea in Arabidopsis
Source: PLoS One. 2012 Apr 26;7(4):e35995. doi: 10.1371/journal.pone.0035995 (PMC3338558; doi:10.1371/journal.pone.0035995)
Supplement: Figure S1 — Significantly over-represented GO terms in the genes upregulated in 35S:ERF5 or 35S:ERF6 plants. Directed acyclic hierarchical graph (DAG) of significantly over-represented gene ontology (GO) terms in the genes upregulated in 35S:ERF5 or 35S:ERF6 plants. The DAG was generated using FatiGO (http://babelomics.bioinfo.cipf.es). GO terms in red are significantly over-represented in the dataset. (PDF) [file pone.0035995.s001.pdf]

Figure S1. Directed acyclic hierarchical graph (DAG) of gene ontology terms significantly over-represented in the genes upregulated in *35S:ERF5* or *35S:ERF6* plants compared to the rest of the Arabidopsis genome. The DAG was generated using FatiGO (http://babelomics.bioinfo.cipf.es). GO terms in red are significantly over-represented in the dataset

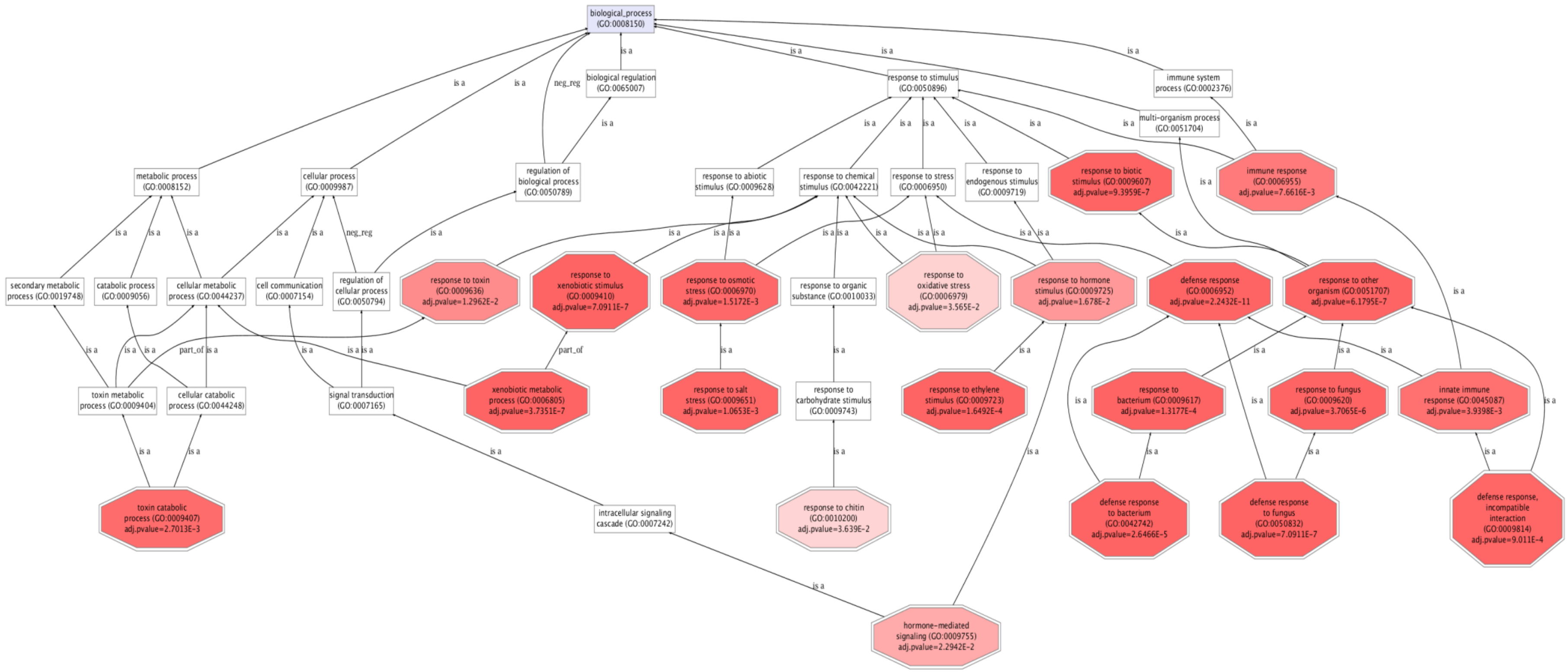

GO biological process (levels from 3 to 9)
