# Supplementary material for: ERF5 and ERF6 Play Redundant Roles as Positive Regulators of JA/Et-Mediated Defense against Botrytis cinerea in Arabidopsis
Source: PLoS One. 2012 Apr 26;7(4):e35995. doi: 10.1371/journal.pone.0035995 (PMC3338558; doi:10.1371/journal.pone.0035995)

Figure S2. The *erf5 erf6* double mutant shows reduced expression of *ERF5* and *ERF6*. Relative accumulation of *ERF5* or *ERF6* mRNA was measured by qRT-PCR in ten-day old seedlings. Relative Quantitation (RQ) values were calculated after normalization to *PEX4* expression levels. Each value is the mean of three technical replicates and the data are representative of three independent experiments. The error bars represent RQ_{MIN} and RQ_{MAX} and constitute the acceptable error level for a 95% confidence level according to Student's *t*-test.

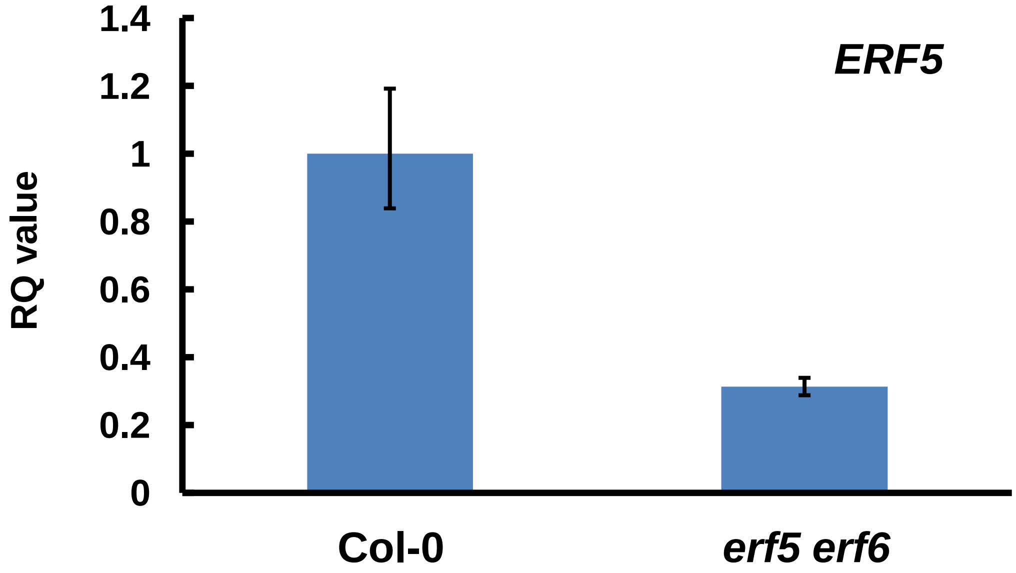

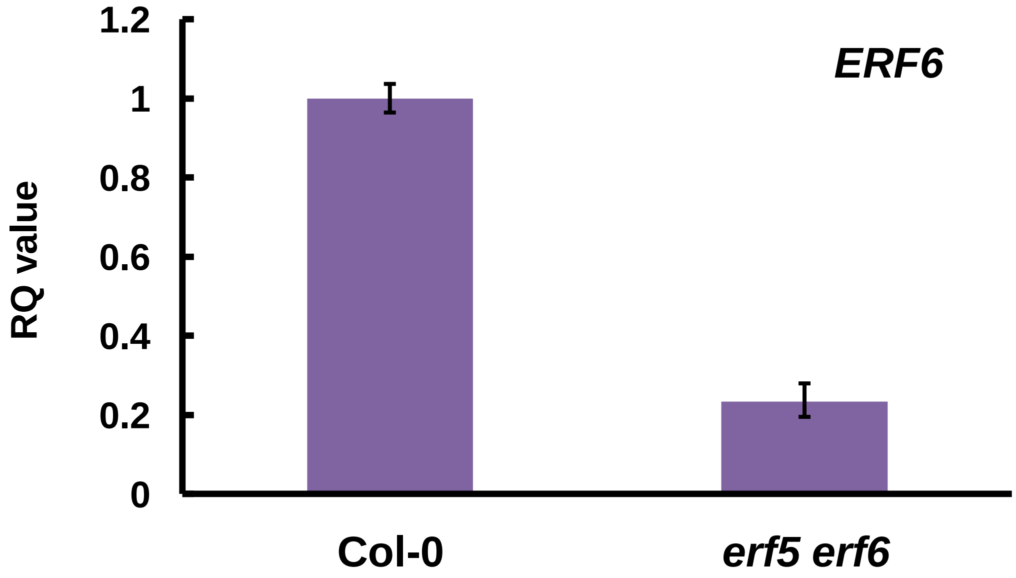

Supplement: Figure S2 — The erf5 erf6 double mutant shows reduced expression of ERF5 and ERF6 . Relative accumulation of ERF5 or ERF6 mRNA was measured by qRT-PCR in ten-day old seedlings. Relative Quantitation (RQ) values were calculated after normalization to PEX4 expression levels. Each value is the mean of three technical replicates and the data are representative of three independent experiments. (PDF) [file pone.0035995.s002.pdf]
