# Supplementary material for: ERF5 and ERF6 Play Redundant Roles as Positive Regulators of JA/Et-Mediated Defense against Botrytis cinerea in Arabidopsis
Source: PLoS One. 2012 Apr 26;7(4):e35995. doi: 10.1371/journal.pone.0035995 (PMC3338558; doi:10.1371/journal.pone.0035995)
Supplement: Table S1 — Promoter motif enrichment analysis of genes significantly upregulated in 35S:ERF5 plants. (PDF) [file pone.0035995.s003.pdf]

Table S1. Promoter motif enrichment analysis of genes significantly upregulated in *35S:ERF5* plants

Motif length (bp)	Identifier	Ratio[a]	Occ[b]	Exp occ[c]	Occ *p*[d]	Occ *e*[e]	Z score[f]	Matching sequences[g]
5	ccgcc|ggcgg	2.3	20	8.76	0.00077	7.90E-01	3.8	16
5	gccca|tgggc	2.2	47	21.72	1.70E-06	1.80E-03	5.42	19
5	cagcc|ggctg	2.1	42	19.87	1.00E-05	1.00E-02	4.97	24
5	gagga|tcctc	1.8	39	21.15	0.00032	3.30E-01	3.88	23
5	ccacc|ggtgg	1.8	42	23.83	0.00048	4.90E-01	3.72	24
5	agaga|tctct	1.4	99	70.85	0.0009	9.20E-01	3.34	33
6	gcagcc|ggctgc	5.1	15	2.96	5.70E-07	2.30E-03	6.99	13
6	gccgcc|ggcggc[h]	5.0	9	1.8	0.00011	4.40E-01	5.37	9
6	ggaccc|gggtcc	4.4	10	2.27	0.00013	5.20E-01	5.13	9
6	cagccg|cggctg	3.8	12	3.13	0.00011	4.20E-01	5.01	8
6	agccgc|gcggct	3.8	11	2.92	0.00024	9.40E-01	4.72	9
6	agccca|tgggct	3.3	26	7.78	2.10E-07	8.40E-04	6.54	10
6	gccacc|ggtggc	3.2	15	4.76	0.00013	5.40E-01	4.7	13
7	agccgcc|ggcggct[i]	13.9	8	0.57	1.80E-07	2.30E-03	9.8	8
7	cagccgc|gcggctg	13.6	6	0.44	7.20E-06	9.50E-02	8.35	4
7	ccggccc|gggccgg	12.8	5	0.39	5.60E-05	7.40E-01	7.36	2
7	agcagcc|ggctgct	11.5	11	0.96	6.30E-09	8.40E-05	10.28	10
7	ggaccca|tgggtcc	7.4	7	0.95	6.10E-05	8.10E-01	6.2	7
7	accagcc|ggctggt	7.3	9	1.23	5.80E-06	7.70E-02	7.01	7
7	cgttgga|tccaacg	6.5	13	2.01	2.20E-07	2.90E-03	7.75	12
7	ggtgtac|gtacacc	5.8	9	1.56	3.80E-05	5.00E-01	5.95	3
7	ccaacgg|ccgttgg	5.6	9	1.6	4.50E-05	6.00E-01	5.85	7
7	gggtcca|tggaccc	5.5	9	1.65	5.80E-05	7.60E-01	5.72	8
7	aagccca|tgggctt	4.2	12	2.89	5.00E-05	6.70E-01	5.36	8
7	atcaacc|ggttgat	3.9	17	4.38	3.70E-06	5.00E-02	6.03	15
7	agataga|tctatct	2.8	20	7.23	6.80E-05	9.00E-01	4.75	13
8	agcagccg|cggctgct	26.2	4	0.15	2.00E-05	6.00E-01	9.85	3
8	ccgttgga|tccaacgg	18.2	7	0.39	1.80E-07	5.40E-03	10.66	6
8	tggaccca|tgggtcca	15.3	6	0.39	3.60E-06	1.10E-01	8.95	6
8	gtacacca|tggtgtac	13.4	8	0.6	2.40E-07	7.10E-03	9.58	3
8	aaccagcc|ggctggtt	13.3	6	0.45	7.80E-06	2.40E-01	8.28	5
8	atccaacg|cgttggat	11.8	10	0.85	2.40E-08	7.20E-04	9.95	9
8	gtgtacta|tagtacac	9.5	7	0.74	1.30E-05	3.80E-01	7.27	3
8	aggttgat|atcaacct	7.9	9	1.14	3.30E-06	9.90E-02	7.35	9

[a] Observed/expected occurrence ratio
[b] Observed occurrences
[c] Expected occurrences
[d] Occurrence probability (binomial)
[e] *e*-value for occurrences (binomial)
[f] Z score(Gaussian approximation)
[g] Number of genes which contain at least one occurrence
[h] GCC core
[i] GCC box
