# Supplementary material for: ERF5 and ERF6 Play Redundant Roles as Positive Regulators of JA/Et-Mediated Defense against Botrytis cinerea in Arabidopsis
Source: PLoS One. 2012 Apr 26;7(4):e35995. doi: 10.1371/journal.pone.0035995 (PMC3338558; doi:10.1371/journal.pone.0035995)
Supplement: Table S2 — Promoter motif enrichment analysis of genes significantly upregulated in 35S:ERF6 plants. (PDF) [file pone.0035995.s004.pdf]

Table S2. **Promoter motif enrichment analysis of genes significantly upregulated in *35S:ERF6* plants**

Motif length (bp)	Identifier	Ratio[a]	Occ[b]	Exp occ[c]	Occ *p*[d]	Occ *e*[e]	Z score[f]	Matching sequences[g]
5	ccgcc|ggcgg	2.3	27	11.7	8.60E-05	8.90E-02	4.45	20
5	gcagc|gctgc	2.1	36	17.0	3.90E-05	4.00E-02	4.6	20
5	gagga|tcctc	1.9	50	26.6	3.40E-05	3.50E-02	4.53	24
5	gaaga|tcttc	1.5	120	80.9	2.90E-05	2.90E-02	4.3	39
5	acaag|cttgt	1.5	121	83.4	6.30E-05	6.40E-02	4.1	37
5	gagaa|ttctc	1.4	128	90.7	0.00012	1.30E-01	3.9	37
5	atgga|tccat	1.4	97	68.9	0.0008	8.20E-01	3.4	37
5	aagaa|ttctt	1.3	242	193.1	0.00038	3.90E-01	3.5	39
6	gccgcc|ggcggc[h]	5.4	12	2.2	4.00E-06	1.60E-02	6.6	12
6	agccgc|gcggct	3.9	13	3.3	4.40E-05	1.80E-01	5.3	11
6	gcagcc|ggctgc	3.7	13	3.5	7.70E-05	3.10E-01	5.1	11
6	agcagc|gctgct	3.0	16	5.3	0.00014	5.70E-01	4.6	13
6	cacaag|cttgtg	2.1	36	17.4	6.10E-05	2.50E-01	4.5	23
6	agaaga|tcttct	1.8	52	29.3	9.60E-05	3.90E-01	4.2	29
6	aattag|ctaatt	1.6	63	38.5	0.00018	7.20E-01	4.0	31
6	aaatta|taattt	1.4	150	105.7	2.80E-05	1.10E-01	4.3	37
6	aagaaa|tttctt	1.4	118	83.6	0.00022	8.90E-01	3.8	39
7	agccgcc|ggcggct[i]	14.2	10	0.7	4.50E-09	6.20E-05	11.1	10
7	agcagcc|ggctgct	7.1	8	1.1	2.50E-05	3.40E-01	6.5	7
7	accagcc|ggctggt	5.7	9	1.6	4.10E-05	5.70E-01	5.9	7
7	aattagg|cctaatt	3.3	17	5.2	2.80E-05	3.90E-01	5.2	14
7	aaacaaa|tttgttt	1.7	68	40.6	5.30E-05	7.30E-01	4.3	33
8	agccgccg|cggcggct	37.1	4	0.1	5.20E-06	1.70E-01	11.9	4
8	agccgccc|gggcggct	33.5	4	0.1	7.70E-06	2.50E-01	11.2	4
8	ggcggcta|tagccgcc	28.2	5	0.2	1.30E-06	4.10E-02	11.5	5
8	agcagccg|cggctgct	24.0	4	0.2	2.80E-05	9.20E-01	9.4	3
8	aaccagcc|ggctggtt	11.7	7	0.6	3.30E-06	1.10E-01	8.3	6
8	agccatca|tgatggct	7.2	8	1.1	2.20E-05	7.10E-01	6.5	8
8	tagataga|tctatcta	5.8	13	2.2	7.10E-07	2.30E-02	7.2	11
8	taatttca|tgaaatta	3.5	19	5.5	4.90E-06	1.60E-01	5.8	16

[a] Observed/expected occurrence ratio
[b] Observed occurrences
[c] Expected occurrences
[d] Occurrence probability (binomial)
[e] *e*-value for occurrences (binomial)
[f] Z score(Gaussian approximation)
[g] Number of genes which contain at least one occurrence
[h] GCC core
[i] GCC box
