# Supplementary material for: ERF5 and ERF6 Play Redundant Roles as Positive Regulators of JA/Et-Mediated Defense against Botrytis cinerea in Arabidopsis
Source: PLoS One. 2012 Apr 26;7(4):e35995. doi: 10.1371/journal.pone.0035995 (PMC3338558; doi:10.1371/journal.pone.0035995)
Supplement: Table S3 — Fold induction values of ERF5 , ERF6 , ERF1 and ORA59 in response to ethylene and jasmonic acid treatment. Fold change in transcript level observed in 7-d old wild-type Col-0 plants treated with 10 µM ACC or 10 µM MeJA for 0.5, 1 or 3 h. Microarray data from the AtGenExpress project with the TAIR submission number ME00334 (ACC) and ME00337 (MeJA) [48]. Values obtained from the eFP Browser on the Botany Array Resource (BAR) [49]. (PDF) [file pone.0035995.s005.pdf]

Table S3. Fold induction values of *ERF5*, *ERF6*, *ERF1* and *ORA59* in response to ethylene and jasmonic acid treatment. Fold change in transcript level observed in 7-d old wild-type Col-0 plants treated with 10 µM ACC or 10 µM MeJA for 0.5, 1 or 3 h. Microarray data from the AtGenExpress project with the TAIR submission number ME00334 (ACC) and ME00337 (MeJA) [48]. Values obtained from the eFP Browser on the Botany Array Resource (BAR) [49].

AGI number	Gene description	ACC 0.5 h	ACC 1 h	ACC 3 h	MeJA 0.5 h	MeJA 1 h	MeJA 3 h
At5g47230	ERF5	1.77	1.63	1.14	1.39	1.12	0.91
At4g17490	ERF6	0.92	1.96	1.24	2.51	3.17	0.86
At3g23240	ERF1	0.68	2.03	2.14	2.48	2.73	1.4
At1g06160	ORA59	1.41	3.88	1.75	7.59	6.57	1.32
