# Supplementary material for: ERF5 and ERF6 Play Redundant Roles as Positive Regulators of JA/Et-Mediated Defense against Botrytis cinerea in Arabidopsis
Source: PLoS One. 2012 Apr 26;7(4):e35995. doi: 10.1371/journal.pone.0035995 (PMC3338558; doi:10.1371/journal.pone.0035995)
Supplement: Table S4 — Fold induction values of ERF5 , ERF6 , ERF1 and ORA59 in response to Botrytis cinerea infection. Fold change in transcript level observed in 4-week old wild-type Col-0 plants inoculated with B. cinerea spores at 18 or 48 h post-inoculation. Microarray data from the AtGenExpress project with the TAIR submission number ME00341. Values obtained from the eFP Browser on the Botany Array Resource (BAR) [49]. (PDF) [file pone.0035995.s006.pdf]

Table S4. Fold induction values of *ERF5*, *ERF6*, *ERF1* and *ORA59* in response to *Botrytis cinerea* infection. Fold change in transcript level observed in 4-week old wild-type Col-0 plants inoculated with *B. cinerea* spores at 18 or 48 h post-inoculation. Microarray data from the AtGenExpress project with the TAIR submission number ME00341. Values obtained from the eFP Browser on the Botany Array Resource (BAR) [49].

AGI number	Gene description	18 h	48 h
At5g47230	ERF5	1.3	1.35
At4g17490	ERF6	1.15	1.25
At3g23240	ERF1	4.31	11.52
At1g06160	ORA59	4.12	1.93
